# Characterization of *Yersinia pestis* Interactions with Human Neutrophils *In vitro*

**DOI:** 10.3389/fcimb.2017.00358

**Published:** 2017-08-09

**Authors:** Sophia C. Dudte, B. Joseph Hinnebusch, Jeffrey G. Shannon

**Affiliations:** Laboratory of Zoonotic Pathogens, Rocky Mountain Laboratories, National Institute of Allergy and Infectious Diseases, National Institutes of Health Hamilton, MT, United States

**Keywords:** *Yersinia pestis*, plague, bubonic, neutrophil, polymorphonuclear, intracellular, activation

## Abstract

*Yersinia pestis* is a gram-negative, zoonotic, bacterial pathogen, and the causative agent of plague. The bubonic form of plague occurs subsequent to deposition of bacteria in the skin by the bite of an infected flea. Neutrophils are recruited to the site of infection within the first few hours and interactions between neutrophils and *Y. pestis* have been demonstrated *in vivo*. In contrast to macrophages, neutrophils have been considered non-permissive to *Y. pestis* intracellular survival. Several studies have shown killing of the vast majority of *Y. pestis* ingested by human neutrophils. However, survival of 10–15% of *Y. pestis* after phagocytosis by neutrophils is consistently observed. Furthermore, these surviving bacteria eventually replicate within and escape from the neutrophils. We set out to further characterize the interactions between *Y. pestis* and human neutrophils by (1) determining the effects of known *Y. pestis* virulence factors on bacterial survival after uptake by neutrophils, (2) examining the mechanisms employed by the neutrophil to kill the majority of intracellular *Y. pestis*, (3) determining the activation phenotype of *Y. pestis*-infected neutrophils, and (4) characterizing the *Y. pestis*-containing phagosome in neutrophils. We infected human neutrophils *in vitro* with *Y. pestis* and assayed bacterial survival and uptake. Deletion of the caf1 gene responsible for F1 capsule production resulted in significantly increased uptake of *Y. pestis*. Surprisingly, while the two-component regulator PhoPQ system is important for survival of *Y. pestis* within neutrophils, pre-induction of this system prior to infection did not increase bacterial survival. We used an IPTG-inducible mCherry construct to distinguish viable from non-viable intracellular bacteria and determined the association of the *Y. pestis*-containing phagosome with neutrophil NADPH-oxidase and markers of primary, secondary and tertiary granules. Additionally, we show that inhibition of reactive oxygen species (ROS) production or Src family kinases increased survival of intracellular bacteria indicating that both ROS and granule-phagosome fusion contribute to neutrophil killing of *Y. pestis*. The data presented here further our understanding of the *Y. pestis* neutrophil interactions and suggest the existence of still unknown virulence factors involved in *Y. pestis* survival within neutrophils.

## Introduction

*Yersinia pestis*, causative agent of bubonic plague, is a zoonotic pathogen with a complicated life cycle primarily involving rodents. Rodent-to-rodent transmission occurs via the flea vector, which can also result in incidental transmission to humans. Human infection results from deposition of *Y. pestis* in the dermis via the bite of an infected flea. There, *Y. pestis* faces a robust innate immune response that must be overcome, evaded, or subverted for infection to be established.

Neutrophils are a critical component of the mammalian innate immune system and act as the first line of defense against invading pathogens. Oxidative killing of bacteria is a hallmark of neutrophils and refers to the NADPH-oxidase dependent respiratory burst. This respiratory burst results in the production of a variety of reactive oxygen species (ROS) including superoxide, hydrogen peroxide, and hypochlorous acid. Neutrophils also use non-oxidative killing mechanisms mediated by delivery of antimicrobial peptide and protease-containing vesicles termed granules to the phagosomes. Granules are divided into four subtypes: primary (1°, azurophilic), secondary (2°, specific), tertiary (3°, gelatinase), and secretory granules (Pham, 2006). Thus, armed with such an array of antimicrobial weapons, neutrophils represent a significant barrier to any invading pathogen.

*Yersinia pestis* has significant interactions *in vivo* with neutrophils (Shannon et al., 2013). Within 30 min after intradermal *Y. pestis* infection, neutrophils are recruited to the skin, and by 4 h, massive recruitment of neutrophils to infected tissue is evident by intravital microscopy (Shannon et al., 2013). *Y. pestis* are rapidly phagocytosed by neutrophils *in vitro*, and the majority of bacteria taken up by neutrophils are killed. However, roughly 15% of the intracellular population not only survive within neutrophil phagosomes, but continue to replicate within the compartment and eventually escape the neutrophil (Spinner et al., 2013). This raises two fundamental questions regarding the *Y. pestis*-neutrophil interaction. First: what *Y. pestis* virulence factors are critical to intracellular survival within human neutrophils? And second: by which mechanism do neutrophils kill intracellular *Y. pestis*?

*Yersinia pestis* maintains a virulence plasmid, pCD1 that encodes a type three secretion system (T3SS) and a variety of secreted effector proteins (Yops) (Viboud and Bliska, 2005). Expression of the T3SS and effector Yops are thermoregulated with maximal expression at the mammalian host temperature 37°C and minimal expression at 21–28°C (Bacon and Burrows, 1956; Cavanaugh and Randall, 1959; Spinner et al., 2008), the temperature of the flea vector. Expression of the T3SS reduces uptake of *Y. pestis* by human neutrophils from 90 to 70% but a majority of bacteria are unable to evade phagocytosis (Spinner et al., 2008). Interestingly, the effector Yops completely inhibit the respiratory burst of human PMNs, yet the intracellular survival of T3SS-positive and negative strains of *Y. pestis* are indistinguishable (Spinner et al., 2008). Thus, *Y. pestis* is uniquely resistant to or can otherwise suppress the effects of the respiratory burst by an unknown T3SS-independent mechanism.

There are many *Y. pestis* virulence factors that could potentially enhance the ability of the bacteria to survive within human neutrophils. In this study, we investigated the role of *Y. pestis* F1 protein capsule, the transcriptional regulator OxyR, the attachment and invasion locus protein Ail, and the two-component regulatory system PhoPQ. F1 is a capsular protein that forms an envelope around the bacteria when grown at 37°C and has a moderate inhibitory effect on phagocytosis by macrophages (Du et al., 2002). OxyR is a dedicated ROS sensing system that aids aerobic organisms in dealing with oxidative stress (Dubbs and Mongkolsuk, 2012). *Y. pestis* encounters extreme oxidative stress within the neutrophil phagosome during the respiratory burst, thus we wanted to determine if OxyR influenced the ability of intracellular *Y. pestis* to survive. Ail is an outer membrane protein and essential virulence factor involved in attachment and invasion of mammalian host epithelial cells and monocytes. Ail has been implicated in neutrophil tropism of *Y. pestis in vivo* (Merritt et al., 2015). Ail is also crucial for *Y. pestis* resistance to complement-mediated lysis and its ability to avoid extensive neutrophil recruitment to sites of infection (Bartra et al., 2008; Hinnebusch et al., 2011). PhoPQ is a two-component regulatory system used by Gram-negative bacteria to sense and respond to signals indicative of the intraphagosomal space, such as antimicrobial peptides, low pH, low Mg^2+^, and low Ca^2+^ (Groisman, 2001). There are two promoters for the *phoPQ* operon—the inducible promoter that is active during low Mg^2+^ conditions and a constitutive promoter that does not require or respond to regulation by PhoP. O'Loughlin et al. determined that PhoPQ increases intracellular survival of *Y. pestis* in human neutrophils (O'Loughlin et al., 2009).

The contribution of these virulence factors was examined in *Y. pestis* strain KIM6+. This strain is highly attenuated *in vivo* due to the lack of the pCD1 virulence plasmid that encodes the T3SS and associated effectors. We chose this strain because previous studies showed that while the T3SS effectors inhibit phagocytosis of *Y. pestis* by neutrophils, they do not affect survival of the bacteria after phagocytosis (Spinner et al., 2008, 2013). Furthermore, our broad interest is in the fate of *Y. pestis* immediately following flea-transmission, at which time expression of the T3SS is downregulated.

## Methods

### Bacterial strains and culture conditions

*Yersinia pestis* was grown from freezer stock overnight in Brain Heart Infusion broth (BHI, Teknova) at 28°C. The following day, the culture was sub-cultured into fresh medium and grown again overnight at 37°C. Bacteria were sub-cultured at 37°C for 2–4 h prior to neutrophil experiments. Low Mg^2+^ medium was based on a medium described by Garcia Vescovi et al. (1996). The medium contained a final concentration of 128 μM Mg^2+^. By titration we determined this to be the minimum Mg^2+^ concentration that supported *Y. pestis* growth and induced PhoP-regulated genes (data not shown). The same medium containing 1,000 μM Mg^2+^ was used for uninduced controls. For growth in low pH medium, BHI broth was adjusted to pH 6.0 by addition of 1N HCl. Strain KIM6+ (pgm+, pCD1-negative) served as the parental strain in these experiments. The KIM6+ Δ*oxyR* mutant was kindly provided by Erickson et al. (2011). The KIM6+ Δ*ail* strain was constructed as described by Bartra et al. (2008). The KIM6+ Δ*phoP* strain was constructed as described by Rebeil et al. (2004). The KIM6+ Δ*caf* 1 mutant was made using the phage λ Red recombinase system (Datsenko and Wanner, 2000) modified for use with *Yersinia pestis* (Derbise et al., 2003). A strain of KIM6+ containing a pTac-mCherry plasmid was used for immunofluorescence experiments. The pTac-mCherry plasmid was constructed by amplifying the mCherry gene from the pmCherry vector (Clontech, Mountain View, CA) by PCR and ligation into the pTAC-MAT-Tag-2 plasmid (Sigma, St. Louis, MO). A strain of KIM6+ transformed with the pMMB67 plasmid (kindly provided by J. Bliska, Stonybrook University) encoding mCherry under control of the *ugd* promotor making it PhoP-regulated was used to confirm PhoP-inducing conditions.

### Isolation of human neutrophils

Human neutrophils used in this study were generously provided by the laboratory of Dr. Frank DeLeo, Rocky Mountain Laboratories, NIAID, NIH. Human heparinized blood was obtained from healthy donors in accordance with a protocol approved by the Institutional Review Board for Human Subjects of NIAID (Bethesda, MD, USA). Written informed consent was obtained from each participant. Human neutrophils were isolated and purified as described (Kobayashi et al., 2002).

### Phagocytosis and intracellular survival assays

Survival curves were generated as follows. Wells of a flat bottom 96 well plate were coated and bacteria were opsonized with 20% autologous human serum in Phosphate-Buffered Saline (PBS, Gibco Life Technologies, Grand Island, NY) for 30 min at 37°C. For experiments with the Δail mutant strain, bacteria were opsonized with 20% heat-inactivated autologous human serum due to the susceptibility of this strain to complement-mediated killing. Bacteria were counted in a Petroff-Hauser chamber and readjusted to 5 × 10^7^/mL. Aliquots of the innocula were serially diluted, plated on blood agar and incubated for 24–48 h at 28°C, and the colonies enumerated to verify the original concentration. Neutrophils were seeded at 2.5 × 10^6^/mL, total of 1 × 10^6^ per well, for 1 h at 37°C in RPMI 1640 +L-glutamine, -phenol red (Gibco Life Technologies, Grand Island, NY) buffered with 10 mM HEPES (MP Biomedicals, Solon, Ohio). When included, 10 μM Diphenyleneiodonium (DPI, Sigma Aldrich), or 30 μM PP1 (Adipogen) was added to the neutrophils 30 min prior to infection. Neutrophils were infected with KIM6+ at an MOI of 5 and the plates were centrifuged at 100 *rcf* for 5 min to synchronize phagocytosis. Plates were incubated for 10, 60, 120, and 240 min at 37°C, 5% CO_2_. At each time point, the plate was removed from the incubator and immediately chilled on ice. Neutrophils were lysed in 0.02% TritonX-100 in PBS for 5 min on ice. The bacteria and neutrophils were triturated in the well. Neutrophil lysates were diluted 1:10 in PBS and added to Lysing Matrix Z bead beating tubes (MP Biomedicals, Solon, OH) and pulsed at speed setting 4 in a FastPrep FP120 Cell Disruptor (Thermo Savant, Carslbad, CA) for 5 s to ensure total lysis of the neutrophils and release of the bacteria. Serial dilutions were plated on blood agar plates, incubated at 28°C, and colonies were enumerated after 24–48 h. Percent survival was calculated by dividing the concentration of bacteria at each time point by the concentration of the inocula and multiplying by 100.

To determine the percent of bacteria phagocytosed, a separate set of wells was treated with gentamicin (15 μg/mL) at 45 min post infection to eliminate extracellular bacteria. At 60 min, the supernatant containing the gentamicin was aspirated and centrifuged at 10,000 *rcf* to collect any loosely adherent neutrophils or bacteria. The supernatant was removed from the tube. Fresh RPMI was used to wash the remaining adherent cells in the wells of the 96 well plate to wash off any residual gentamicin, and again that wash was pulled off and centrifuged. The pellet was washed one additional time and the bacteria and neutrophils were returned to the appropriate well and processed with TritonX-100 as indicated above. To calculate the percent uptake at 60 min, the concentration of bacteria in the gentamicin treated wells was divided by the concentration of the untreated wells, and multiplied by 100.

### Immunofluorescence assay

*Yersinia pestis* strain KIM6+ was prepared as aforementioned. *S. aureus* strain MnCOP (methicillin-sensitive, kindly provided by Frank DeLeo, Rocky Mountain Laboratories, NIAID, NIH) was grown overnight in BHI at 37°C, washed in PBS, and incubated in 1:500 Alexa 546 in PBS for 20 min at room temperature in the dark. Bacteria were opsonized with 20% normal autologous human serum prior to infection. Neutrophils were prepared as described in the above section on round, fibronectin-coated (200 ug/ml human fibronectin in PBS, 37°C, 30 min) glass coverslips in a 24 well plate. Bacteria were added to neutrophils and the plate centrifuged for 5 min at 100 *rcf*. Plates were then incubated at 37°C, 5% CO_2_ for 15 min. Gentamicin (15 μg/ml final concentration) was added to each well to eliminate extracellular bacteria. After a 15 min gentamicin treatment, the media was removed and replaced with fresh RPMI without gentamicin, but containing 300 μM IPTG to induce mCherry expression in remaining live *Y. pestis*. Cells and bacteria were incubated at 37°C, 5% CO_2_ for an additional 90 min. Medium was removed and the cells fixed with 4% paraformaldehyde in PBS on ice for 15 min. Coverslips were washed twice with PBS and permeabilized with 0.05% tritonx100 in PBS for 5 min at room temperature. Coverslips were blocked for 1 h at room temperature with 10% normal goat serum (ThermoFisher) in PBS. Primary and secondary antibodies were diluted in 2% normal goat serum in PBS. NADPH-oxidase components and granule markers were stained with the following antibodies against human antigens: mouse anti-CD63 (BD Immunocytometry), rabbit anti-neutrophil elastase (Abcam), rabbit anti-p47phox (kindly provide by Frank Deleo, Rocky Mountain Laboratories, NIAID, NIH), mouse anti-gp91phox (Santa Cruz Biotechnology), mouse anti-myeloperoxidase (Abcam), mouse anti-CD66b (BioLegend), and rat anti-*Y. pestis* (convalescent rat serum produced in the Hinnebusch lab). Cells were then washed 4 times with PBS and secondary staining was accomplished with goat anti-mouse, anti-rabbit, or anti-rat conjugated to Alexa488 or Alexa647 dyes for 1 h at room temperature in the dark. Coverslips were washed with PBS 5 times and coverslips were mounted on glass slides with ProLong Diamond + DAPI (ThermoFisher). Z-stacks were obtained on a Zeiss LSM 710 confocal microscope. Images were analyzed and processed for publication using ImageJ software available at http://imagej.net/. Association of the various markers with bacterial phagosomes was also quantified using ImageJ software.

### Flow cytometry

Human neutrophils were infected with *Y. pestis* KIM6+ or *S. aureus* as described above in wells of 96 well plates. Additionally, neutrophils were left uninfected/untreated or stimulated with Phorbol 12-myristate 13-acetate (PMA, 1 μM, Sigma, St. Louis, MO), *E. coli* lipopolysaccharide (LPS, 1 μg/ml, Sigma), or N-formyl-Met-Leu-Phe (fMLP, 10 μM, Sigma) where indicated as positive controls for neutrophil activation. Cells were harvested from wells at 15 min and 4 h post-infection and stained for flow cytometry with a cocktail of fluorescently labeled antibodies against the following markers: CD88-PerCP/Cy5.5 (BioLegend, San Diego, CA), CD66b-Alexa647, CD62L-PETR, CD16-BV786, CD63-PECy7, CD64-Alexa700, CD11b-Alexa488, and CD54-BV421 (all from BD Immunocytometry, San Jose, CA). The fixable viability dye efluor780 (ThermoFisher) was also included and analysis was limited to live, efluor780^low/negative^ cells. After staining, cells were washed with 2% FBS in PBS and resuspended in 2% paraformaldehyde in PBS prior to analysis on a LSR-II flow cytometer (BD Immunocytometry). Data were analyzed using FlowJo 10.2 software.

### Statistics

For phagocytosis assays, statistical differences between KIM6+ pH7.2 and the other conditions was calculated by one-way ANOVA followed by Dunnett's multiple comparison test. For intracellular survival CFU assays, groups were compared using one-way ANOVA followed by Sidak's multiple comparison test. For flow cytometry assays, mean fluorescence intensities (MFI) were compared to uninfected/untreated control MFI by one-way ANOVA followed by Dunnett's multiple comparison test. For all statistical tests, the numbers of replicates in each experiment are described in the Figure Legends.

## Results and discussion

### Increased phagocytosis of Δ*caf1 Y. pestis* by human neutrophils

Our goal was to determine if any of several known or putative *Y. pestis* virulence factors affect bacteria-neutrophil interactions, specifically intracellular survival of *Y. pestis*. Before we could assess intracellular survival, we needed to quantify any potential effects of these factors on phagocytosis of *Y. pestis* by neutrophils. To this end, we infected human peripheral blood neutrophils *in vitro* and quantified phagocytosis of a parental KIM6+ strain of *Y. pestis* and deletion mutants of the *ail, oxyR* or *caf1* genes.

**Figure 1:**
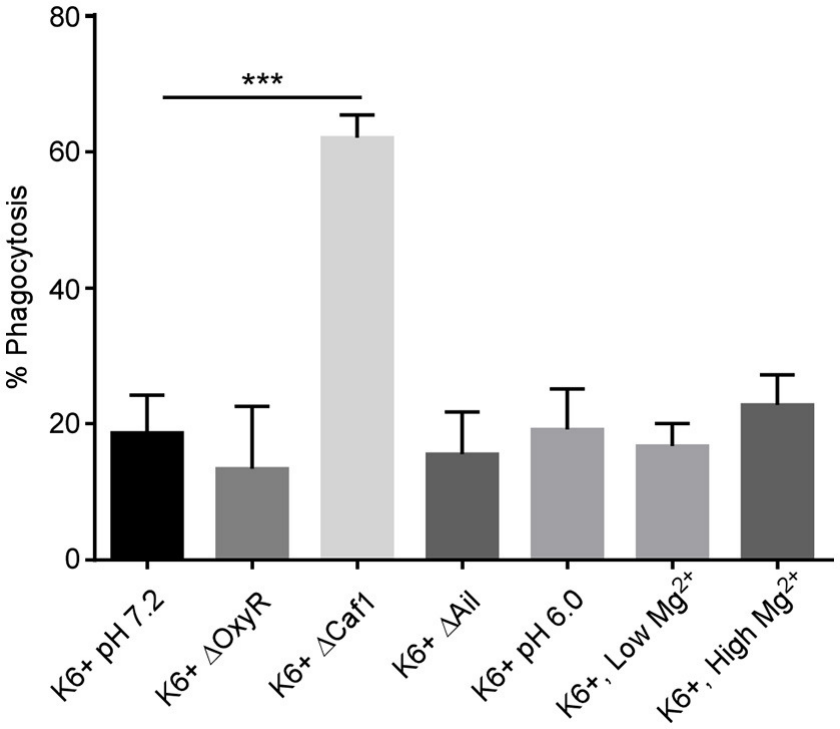
Deletion of the *caf1* gene increases phagocytosis of *Y. pestis* by neutrophils. Human peripheral blood neutrophils were infected *in vitro* with *Y. pestis* KIM6+ or the deletion mutants. Additionally, neutrophils were infected with *Y. pestis* grown at pH6.0 or with low Mg^2+^ to pre-induce PhoPQ expression prior to infection. At 45 min post-infection, gentamicin was added to half the wells to kill extracellular bacteria. At 60 min post-infection all wells were harvested, cells washed, lysed, triturated and plated to quantify CFU. The % phagocytosis was calculated by dividing the number of intracellular CFU from the gentamicin treated wells by the total CFU in the non-treated wells and multiplying by 100. The mean of 3 independent experiments with cells from 3 different donors is shown for each condition. Error bars represent SEM. Statistical differences between KIM6+ pH7.2 and the other conditions were calculated by one-way ANOVA followed by Dunnett's multiple comparison test. ^***^p-value of < 0.001.

We observed an approximately 3-fold increase in phagocytosis of the Δ*caf1* strain compared to wild type KIM6+ (Figure 1). The Δ*oxyR* and Δ*ail* strains were phagocytosed at levels equivalent to *wt* KIM6+. Thus, the F1 protein capsule has a potent anti-phagocytic effect on neutrophils. This observation supports a previous study showing increased phagocytosis of a Δ*caf1* mutant of *Y. pestis* by J774 macrophage-like cell line (Du et al., 2002). It is important to note that in this study we did not test if increased phagocytosis phenotype of the Δ*caf1* strain could be complemented in trans. However, the increased phagocytosis of the Δ*caf1* strain is important to take into account when interpreting the assays of *Y. pestis* survival within neutrophils described below.

### Effects of Ail, Caf1, OxyR, and PhoPQ on survival of *Y. pestis* within human neutrophils

**Figure 2:**
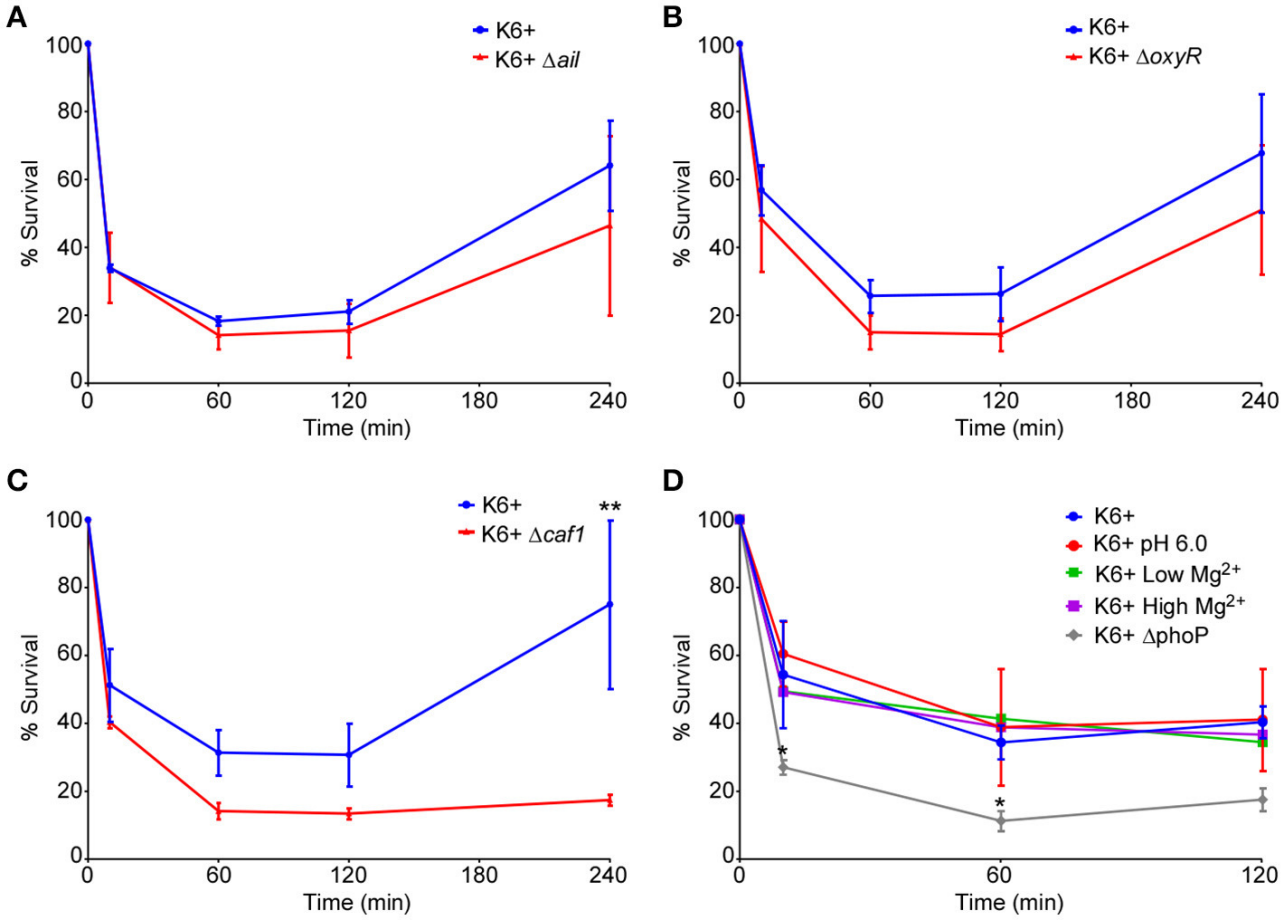
Deletion of *ail, oxyR, caf1*, or pre-induction of PhoPQ do not affect survival of *Y. pestis* within neutrophils. Human neutrophils were infected with KIM6+ or the different deletion mutants or PhoPQ pre-induced bacteria. Cells were lysed, triturated and plated to enumerate CFU at 10, 60, 120, and 240 min post-infection. CFU data are shown as a percentage of the inoculum remaining in each well at the given time point. Each graph represents a separate set of experiments where the mutant/pre-induced strains were assayed side-by-side on the same day with cells from the same donors with the *wt* KIM6+ pH7.2. The deletion mutants **(A)** KIM6+ Δ*ail*, **(B)** KIM6+ Δ*oxyR*, and **(C)** KIM6+ Δ*caf1* were compared to the parental strain. **(D)** KIM6+ grown at pH6.0 or with low Mg^2+^ (128 μM) to pre-induce PhoPQ expression were compared to the same strain grown at pH7.2 or in Mg^2+^-replete medium (High Mg^2+^, 1,000 μM). A KIM6+ ΔphoP mutant strain was included for comparison. Each graph represents the mean of 3 independent experiments. Day-to-day and donor-to-donor variation necessitated running separate *wt* KIM6+ controls alongside each deletion mutant or growth condition tested. Error bars represent SEM. Statistical differences were determined by multiple *T*-tests comparing 2 groups at each time point, corrected for multiple comparisons using Holm-Sidak method. ^*^p < 0.05, ^**^p < 0.01.

To test the potential effects of the subset of virulence factors on intracellular survival of *Y. pestis* within neutrophils, human neutrophils were infected *in vitro* with wt (KIM6+), Δ*ail*, Δ*caf1*, Δ*oxyR* strains of *Y. pestis*. Cells were triturated and plated at 10, 60, 120, and 240 min post-infection to quantify CFUs. Strains lacking the *ail* or *oxyR* genes did not differ in survival in human neutrophils compared to the parent strain (Figures 2A,B). At first glance the Δ*caf1* strain appears to have an intracellular survival defect (Figure 2C), but the decreased CFU numbers recovered is due to greatly increased phagocytosis and subsequent killing of this strain relative to the parental strain as is shown in Figure 1. This was confirmed by microscopy using a live/dead staining based on a technique described by Johnson and Criss (2013) to distinguish between viable and non-viable *Y. pestis*. We found an approximately 2-fold increase in the total number of Δ*caf1* bacteria/neutrophil, but the percentage of viable bacteria did not differ significantly from wt KIM6+ (data not shown).

The two-component signaling system PhoPQ is important for survival of *Y. pestis* within human neutrophils (O'Loughlin et al., 2009). Expression of PhoPQ is down-regulated during growth of *Y. pestis* in standard broth culture medium. However, PhoPQ expression is induced in bacteria isolated from flea midguts (Vadyvaloo et al., 2010). We therefore wanted to test if pre-induction of this regulatory system could increase intracellular survival of *Y. pestis* within neutrophils. We induced PhoPQ expression in *Y. pestis* either by culturing in mildly acidic (pH 6.0) BHI broth or culturing in low Mg^2+^ medium. Both of these conditions have been shown to induce PhoPQ gene expression (Foster and Hall, 1990; Garcia Vescovi et al., 1996). We confirmed that these conditions increased fluorescence in a PhoP-regulated mCherry reporter strain of *Y. pestis* (data not shown). Pre-induction of the PhoPQ system by growth in acidic (pH6.0) or low Mg^2+^ media did not affect overall phagocytosis of *Y. pestis* (Figure 1). We also found that neither of the PhoPQ-inducing conditions increased intracellular survival of *Y. pestis* within neutrophils relative to growth in the normal pH 7.2, Mg^2+^-replete medium (Figure 2D). The KIM6+ Δ*phoP* mutant strain was included to show the assay's sensitivity and confirm the importance of PhoPQ in survival within neutrophils as was previously reported (O'Loughlin et al., 2009).

Taken together, these results show that deletion of *ail* or *oxyR* had no statistically significant effect on survival of *Y. pestis* within neutrophils. Additionally, even though PhoPQ is essential for survival within neutrophils, pre-induction of the PhoPQ system prior to infection of neutrophils did not enhance intracellular survival. These results confirm previous data showing a subset of *Y. pestis* can survive and replicate within human neutrophils in a T3SS-independent manner, but additional work is needed to identify key factors in this phenomenon.

### Oxidative and non-oxidative bactericidal mechanisms contribute to neutrophil killing of *Y. pestis*

**Figure 3:**
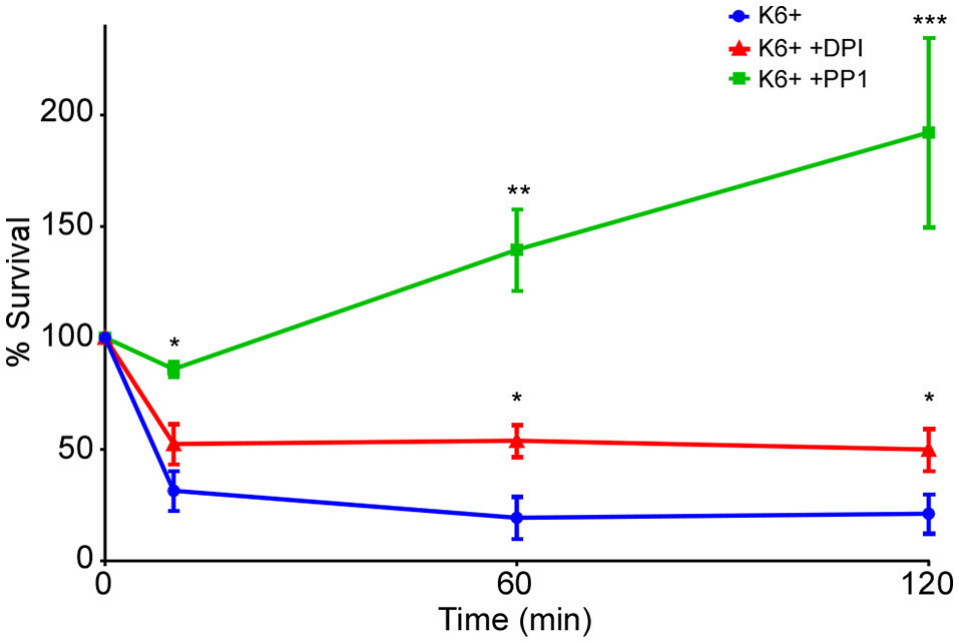
Oxidative and non-oxidative mechanisms contribute to killing of *Y. pestis* by neutrophils. Human neutrophils were mock-treated or treated with the NADPH oxidase inhibitor DPI (10 μM) or the Src kinase inhibitor PP1 (30 μM) 30 min prior to infection with *Y. pestis* KIM6+. Cells were lysed, triturated and plated to enumerate CFU at 10, 60, and 120 min post-infection. Each graph represents the mean of 3 independent experiments. Error bars represent SEM. Statistical differences were determined by multiple *t*-tests comparing 2 groups at each time point, corrected for multiple comparisons using Holm-Sidak method. ^*^p < 0.05, ^**^ < 0.01, ^***^ < 0.001.

To better understand how a subpopulation of *Y. pestis* might survive within neutrophils, we sought to determine the mechanisms used by neutrophils to kill the majority of phagocytosed bacteria. Neutrophils possess oxidative and non-oxidative bactericidal mechanisms. The NADPH phagocyte oxidase complex expressed by neutrophils is essential for oxidative killing mechanisms. DPI inhibits NADPH oxidases and its effects are thought to be limited to the oxidative bactericidal mechanisms with little effect on the non-oxidative. PP1 is an inhibitor of Src family tyrosine kinases, which are essential for neutrophil granule fusion with phagosomes necessary for oxidative and non-oxidative bacterial killing. We tested the effects of both inhibitors on the intracellular survival of *Y. pestis* within neutrophils. PP1 inhibition of Src kinases provided near complete protection of intracellular *Y. pestis*. Inhibition of NADPH-oxidase by DPI significantly increased survival of *Y. pestis*, but not to the level observed for PP1, indicating that both oxidative and non-oxidative bactericidal mechanisms contribute to killing of *Y. pestis* by neutrophils (Figure 3).

### Characterization of the activation and degranulation phenotypes of *Y. pestis*-infected neutrophils

**Figure 4:**
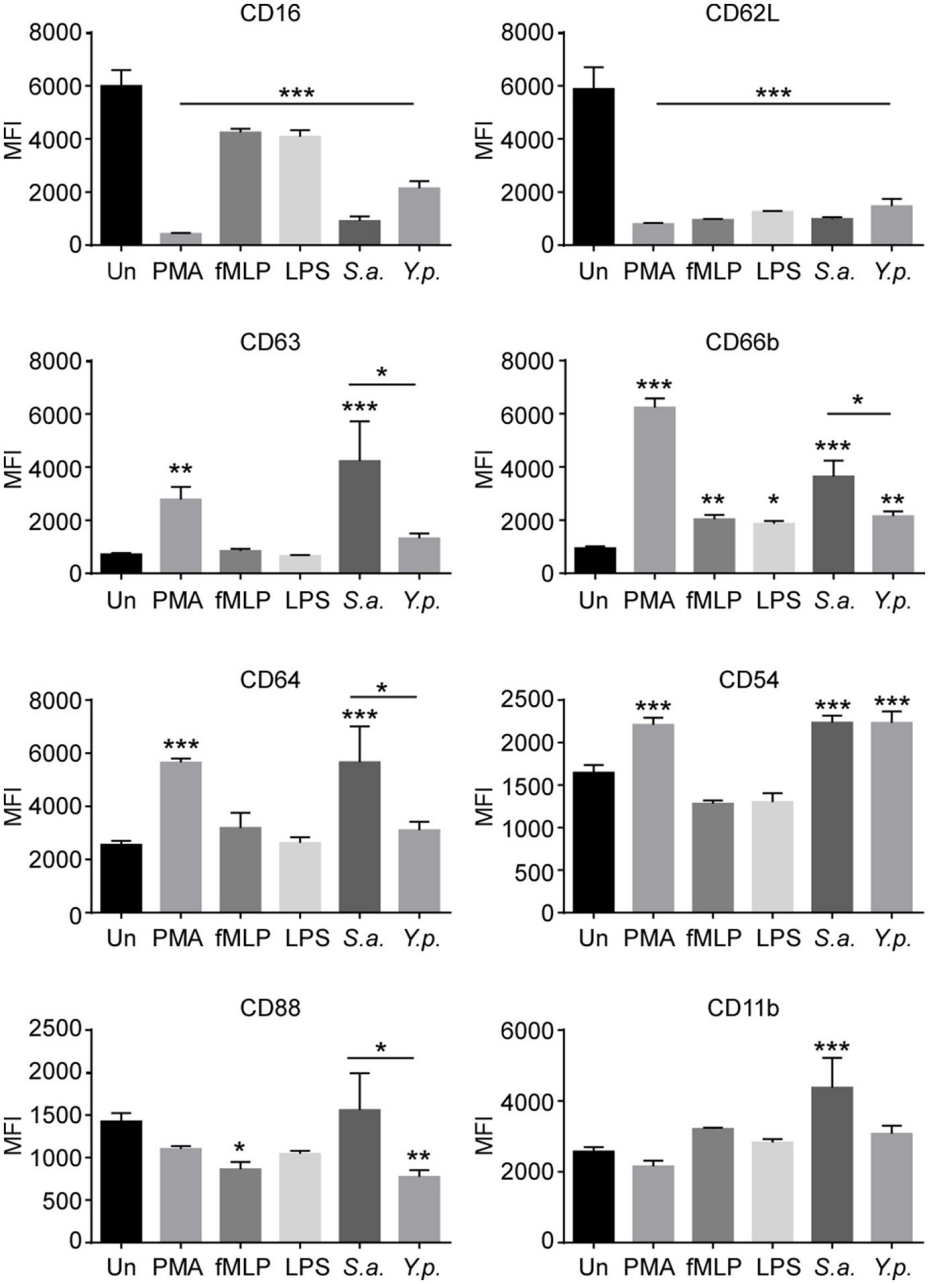
Characterization of neutrophil activation and degranulation phenotype induced by *Y. pestis* infection. Neutrophils were infected with *Y. pestis* KIM6+ (*Y.p*.) or *S. aureus* (*S.a*.) at an MOI of approximately 5. Other samples of neutrophils were activated with PMA, *E. coli* LPS, or fMLP as positive controls. Uninfected/untreated cells served as negative controls. At 4 h post-infection/treatment, neutrophils were harvested and stained for surface expression of eight activation or degranulation markers and analyzed by flow cytometry. The mean fluorescence intensity (MFI) of each surface marker is shown. Error bars represent SD. The results shown are from one experiment done in triplicate and are representative of 3 independent experiments with neutrophils from different donors. MFIs were compared to uninfected/untreated control MFI by one-way ANOVA followed by Dunnett's multiple comparison test. Statistical significance indicated is relative to uninfected/untreated control, unless otherwise indicated. An unpaired *t*-test was used for direct comparison between *S. aureus and Y. pestis* MFI. ^*^p < 0.05, ^**^ < 0.01, ^***^ < 0.001.

Recent studies have uncovered previously unappreciated plasticity in neutrophil differentiation and activation in response to infectious or inflammatory stimuli. Neutrophils have been found to undergo differentiation into pro-inflammatory N1 or anti-inflammatory N2 phenotypes, akin to classically activated M1 vs. alternatively activated M2 macrophages (Martinez and Gordon, 2014; Deniset and Kubes, 2016; de Oliveira et al., 2016). As an example, infection of neutrophils with the pathogenic bacterium *Helicobacter pylori in vitro* induces a pro-inflammatory N1 phenotype similar to neutrophils associated with tumor microenvironments (Whitmore et al., 2017). To determine the effect of *Y. pestis* infection on neutrophil activation phenotype, neutrophils were infected *in vitro* with *Y. pestis*, stimulated with PMA, *E. coli* LPS, or fMLP or left untreated/uninfected. Additionally, we analyzed the response to *Staphylococcus aureus* MnCOP, a methicillin-sensitive, but pathogenic strain shown to be readily phagocytosed by neutrophils and to elicit a strong oxidative burst (Voyich et al., 2005). At 4 h post-infection/treatment, cells were stained for surface expression of neutrophil activation and degranulation markers. Human peripheral blood neutrophils exhibit high surface expression of CD16, or FcγRIII, and CD62L, also known as L-selectin (Figure 4). Typically, neutrophil activation is associated with rapid shedding of CD62L (Walcheck et al., 2006), while loss of CD16 on neutrophils is associated with apoptosis or stimulation (Huizinga et al., 1988; Dransfield et al., 1994). We found that infection with *Y. pestis* or *S. aureus* dramatically reduced surface expression of CD16 and CD62L. PMA proved to be a more potent stimulator of CD16 shedding than *Y. pestis*, whereas *E. coli* LPS and fMLP were both less stimulatory. An average of 50.1 ± 4.8% of neutrophils exhibited a CD16^low^/CD62L^low^ phenotype at 4 h after *Y. pestis* infection compared to 7.1 ± 1.3% of uninfected control neutrophils. There was no indication of the CD16^hi^, CD62L^low^ neutrophil population observed by Whitmore et al. in response to *H. pylori* (Whitmore et al., 2017). Additionally, we did not observe the nuclear hypersegmentation (data not shown) reported after *H. pylori* infection, which is a hallmark of the tumor-associated N1 phenotype (Pillay et al., 2012). Thus, *Y. pestis* appears to induce a more conventional neutrophil activation program, similar to the response to *S. aureus*.

*Yersinia pestis* induced lower surface expression of CD63 and CD66b, markers of 1° and 2° granules, respectively, than *S. aureus*, indicating that *Y. pestis* induced less degranulation (Figure 4). Increased surface expression of CD11b (Mac1, integrin αM) and CD64 (FcγR1) is associated with neutrophil activation (Sengelov et al., 1993; Simms and D'Amico, 1994). *S. aureus* infection induced higher levels of these markers than *Y. pestis*. Both pathogens induced comparable levels of CD54 (intercellular adhesion molecule 1, ICAM1) surface expression, another indicator of neutrophil activation (Roebuck and Finnegan, 1999). Expression of CD88, the C5a anaphylatoxin chemotactic receptor, was significantly reduced in response to fMLP and *Y. pestis*, but not *S. aureus*. Cumulatively, these results show that *Y. pestis* is less stimulatory for neutrophils than *S. aureus*, based on the levels of degranulation markers (CD63 and CD66b) and activation markers (CD11b and CD64); however, both bacteria induced similar levels of CD54 and *Y. pestis* induced significantly more shedding of CD88.

### Association of NADPH oxidase and granule components with *Y. pestis*-containing neutrophil phagosomes

Like other phagocytes, neutrophils ingest bacteria into membrane-bound compartments termed phagosomes. Antimicrobial effectors are contained within neutrophil granules and are delivered to bacteria-containing phagosomes by granule fusion with the phagosomal membrane. Granule fusion can be imaged by immunofluorescence and quantified. As stated before, the majority of *Y. pestis* phagocytosed by neutrophils are killed, but we consistently observe a population of 10–15% of intracellular bacteria that survive. We aimed to identify differences between live and dead *Y. pestis* in intracellular trafficking or granule fusion within neutrophils. To distinguish between live and dead intracellular *Y. pestis*, we used a technique similar to one previously described by Pujol and Bliska in their study showing replication of *Y. pestis* within macrophages (Pujol and Bliska, 2003). We infected neutrophils with a strain of *Y. pestis* possessing a plasmid encoding an IPTG-inducible mCherry fluorescent protein. Extracellular bacteria were killed by gentamicin treatment and mCherry expression induced in the remaining live intracellular bacteria by addition of IPTG to the medium for 90 min. The cells were then fixed and stained for immunofluorescence with antibodies against CD63 (1° granule membrane), gp91phox (2° and 3° granule membrane-bound NADPH oxidase component), p47phox (cytosolic NADPH oxidase component recruited to membranes to form active oxidase complex), or neutrophil elastase (NE, serine protease contained within 1° granules). Staining with anti-*Y. pestis* antiserum permitted identification of mCherry-negative *Y. pestis*. Neutrophils infected with a strain of *S. aureus* known to associate with NADPH oxidase and granule markers served as positive controls. *S. aureus* was labeled with Alexa546 prior to infection, allowing for fluorescence imaging of the bacteria within neutrophils.

**Figure 5:**
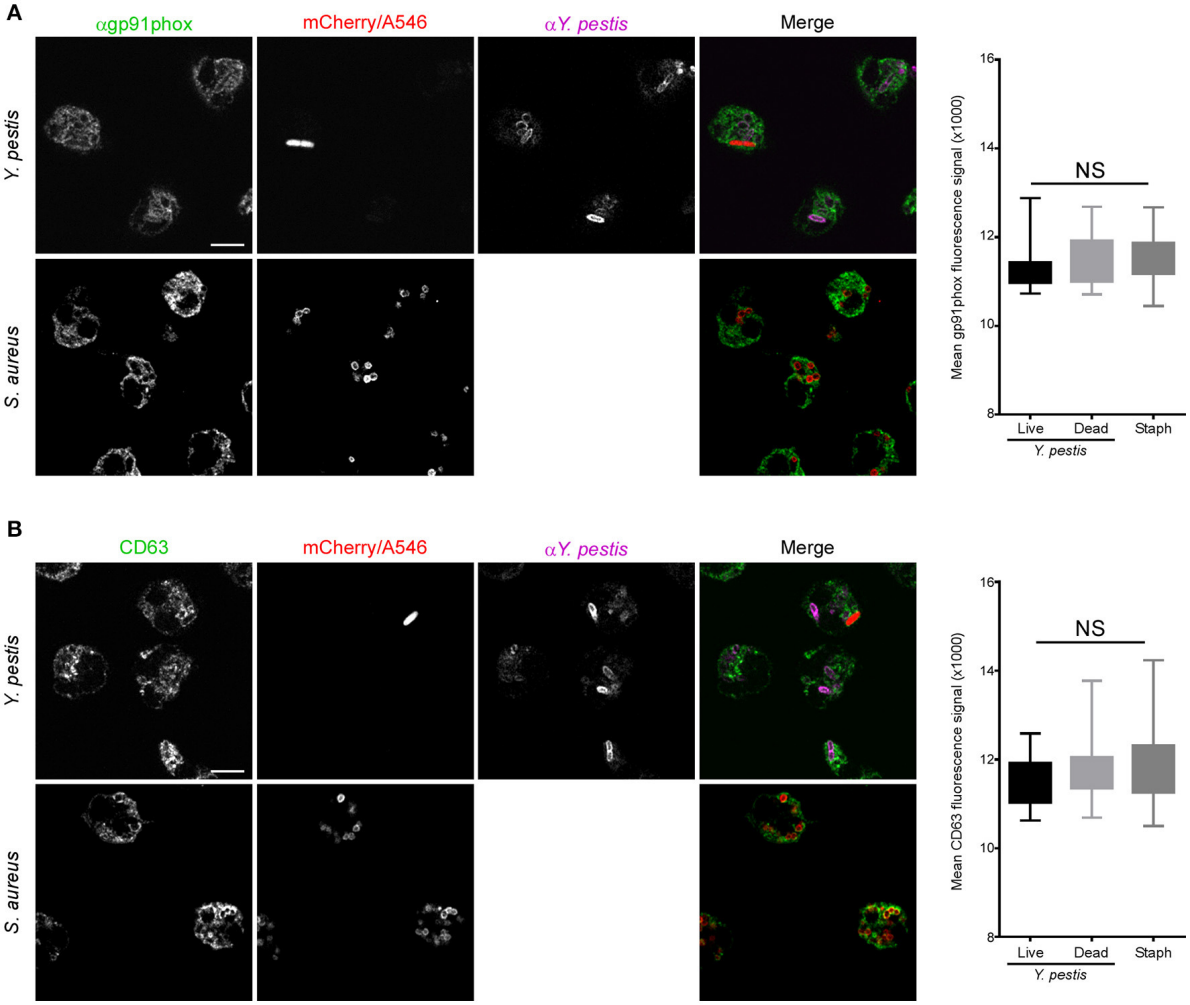
Neutrophil phagosomes containing live or dead *Y. pestis* associate with gp91phox component of NADPH oxidase and CD63. Neutrophils were infected with *Y. pestis* KIM6+ pTac-mCherry or Alexa546-labeled *S. aureus* on fibronectin-coated glass coverslips. At 15 min post-infection gentamicin was added to the medium to kill remaining extracellular bacteria. At 30 min post-infection the medium was removed and replaced with fresh medium without gentamicin, but with 300 μM IPTG. Cells were cultured for an additional 90 min to induce mCherry expression in remaining live *Y. pestis*. Coverslips were fixed, stained for IFA with antibodies against *Y. pestis* and **(A)** gp91phox, or **(B)** CD63, and imaged by confocal microscopy. Live *Y. pestis* were identified by bright mCherry signal. Anti-*Y. pestis* staining was used to identify dead *Y. pestis*. We did not distinguish between live or dead *S. aureus*. Images are representative of 10 separate micrographs for each condition. Scale bar represents 5 μm. The box and whisker plots to the right of each set of panel show combined data for fluorescence intensity surrounding live *Y. pestis*, dead *Y. pestis*, or *S. aureus* phagosomes. Differences in fluorescence intensities were determined by one-way ANOVA followed by Holm-Sidak's multiple comparisons test. The box represents 25–75th percentile, the whiskers represent minimum and maximum values. No statistical difference was found between any of the conditions tested.

**Figure 6:**
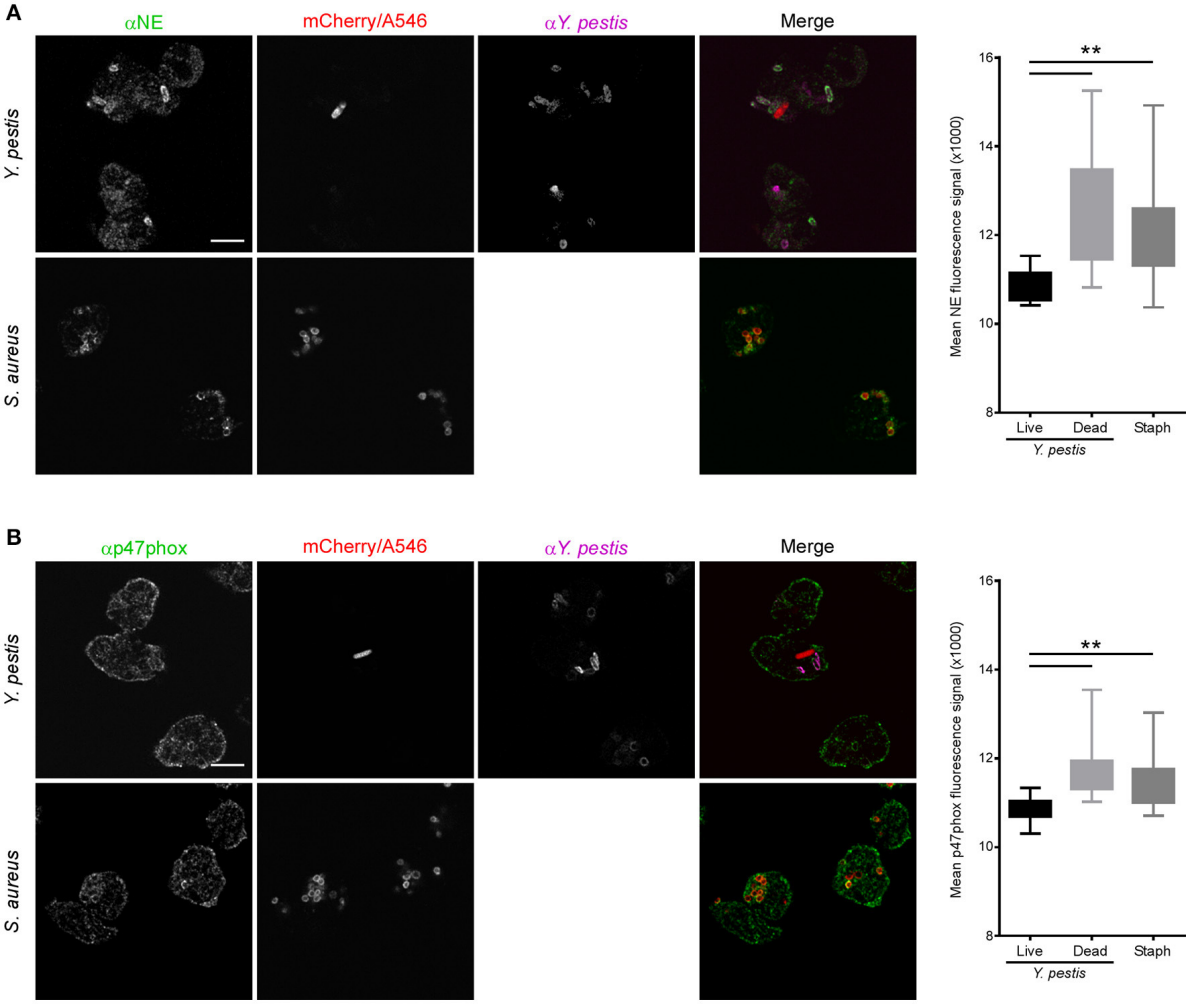
Neutrophil phagosomes containing live *Y. pestis* show reduced association with p47phox and neutrophil elastase. Neutrophils were infected with *Y. pestis* KIM6+ pTac-mCherry or Alexa546-labeled *S. aureus* on fibronectin-coated glass coverslips. At 15 min post-infection gentamicin was added to the medium to kill remaining extracellular bacteria. At 30 min post-infection the medium was removed and replaced with fresh medium without gentamicin, but with 300 μM IPTG. Cells were cultured for an additional 90 min to induce mCherry expression in remaining live *Y. pestis*. Coverslips were fixed, stained for IFA with antibodies against *Y. pestis* and **(A)** neutrophil elastase (NE), or **(B)** p47phox, and imaged by confocal microscopy. Live *Y. pestis* were identified by bright mCherry signal. Anti-*Y. pestis* staining was used to identify dead *Y. pestis*. We did not distinguish between live or dead *S. aureus*. Images are representative of 10 separate micrographs for each condition. Scale bar represents 5 μm. The box and whisker plots to the right of each set of panels show combined data for fluorescence intensity surrounding live *Y. pestis*, dead *Y. pestis* or *S. aureus* phagosomes. Differences in fluorescence intensities were determined by one-way ANOVA followed by Holm-Sidak's multiple comparisons test. The box represents 25–75 percentile, the whiskers represent minimum and maximum values. ^**^p < 0.01.

Phagosomes surrounding live or dead *Y. pestis* stained with anti-gp91phox, similar to *S. aureus* phagosomes, indicating association with membrane-bound components of the NADPH oxidase (Figure 5A). Comparable results were obtained with anti-CD63 staining, indicative of 1° granule fusion with the phagosomal membrane regardless of whether the bacteria were live or dead at the time of fixation (Figure 5B). Interestingly, phagosomes containing live, mCherry+ *Y. pestis* showed reduced staining with anti-NE and anti-p47phox antibodies compared to *S. aureus* or dead *Y. pestis* (Figures 6A,B). These results suggest that a subpopulation of *Y. pestis* can survive by avoiding the toxic components of 1° granules and assembly of the active NADPH oxidase complex. The finding that phagosomes containing live *Y. pestis* associated with the 1° granule membrane marker CD63, but not with NE, a soluble protease contained within 1° granules, was surprising. We cannot currently explain how granule membranes could fuse with the phagosome while the granule contents are excluded. Future work will be aimed at further examination of this phenomenon and determining its mechanism.

In summary, of the virulence factors tested here, only the F1 protein capsule had any appreciable effect on *Y. pestis*-neutrophil interactions *in vitro*. Consistent with a previous observation with macrophages, the F1 protein capsule inhibited phagocytosis of *Y. pestis* by neutrophils. Our results also indicated that both oxidative and non-oxidative mechanisms likely contribute to killing of *Y. pestis* by human neutrophils. Analysis of surface expression of activation and degranulation markers showed the *Y. pestis* infection stimulates neutrophils less than *S. aureus* and does not appear to induce differentiation into any of the alternative activation phenotypes that have been described. We found that the subpopulation of *Y. pestis* that survives phagocytosis by neutrophils avoids complete phagosome association with bactericidal neutrophil components, such as active NADPH oxidase and NE. The mechanisms responsible for evasion of the potent microbicidal activity of neutrophils remain unknown and are a subject of current study.

The importance of survival within and escape from neutrophils in bubonic plague pathogenesis is unknown. It remains possible that uptake by neutrophils *in vivo* is the dead end it has long been assumed to be, and only bacteria that evade neutrophil phagocytosis, either by residing within more permissive macrophages or remaining extracellular throughout the course of infection, go on to disseminate systemically. Recent studies indicate that a small proportion of the *Y. pestis* inoculum can disseminate very quickly after intradermal needle inoculation or flea transmission by a mechanism independent of host cells (Shannon et al., 2013, 2015; Gonzalez et al., 2015). Gonzalez et al. used genetically tagged strains to show that the dermis acts as a significant bottleneck during infection, and only a small number of bacteria make it through to the draining lymph node (dLN) (Gonzalez et al., 2015). The authors propose that the high numbers of neutrophils recruited to the intradermal injection site create a highly inflammatory environment that may restrict the majority of the inoculum to the skin. For the rapid disseminators, what happens immediately after arrival in the dLN is largely unknown. Are they taken up by host cells or do they remain extracellular? Do they encounter neutrophils and, if so, what is the outcome of this interaction? Clearly further study of the roles of neutrophils and other phagocytes in the progression of bubonic plague is warranted.

## Ethics statement

This study was carried out in accordance with the recommendations and guidelines of the Institutional Review Board for Human Subjects, US National Institute of Allergy and Infectious Diseases, National Institutes of Health (protocol number 01-I-N055). All subjects gave written informed consent in accordance with the Declaration of Helsinki.

## Author contributions

SD, BH, and JS designed the study. SD and JS performed the experiments. SD, BH, and JS analyzed the data. SD and JS wrote the paper.

### Conflict of interest statement

The authors declare that the research was conducted in the absence of any commercial or financial relationships that could be construed as a potential conflict of interest.
